# *Neisseria meningitidis* serogroup B causing invasive disease, Italy, 2010–2021

**DOI:** 10.1371/journal.pone.0337446

**Published:** 2025-12-05

**Authors:** Paola Vacca, Fenicia Vescio, Fortunato D’Ancona, Cecilia Fazio, Arianna Neri, Anna Carannante, Luigina Ambrosio, Florigio Romano Lista, Silvia Fillo, Andrea Ciammaruconi, Antonella Fortunato, Paola Stefanelli

**Affiliations:** 1 Department Infectious Diseases, Istituto Superiore di Sanità, Rome, Italy; 2 Environmental and Social Epidemiology Unit, Istituto Superiore di Sanità, Rome, Italy; 3 Scientific Department, Army Medical Centre, Rome, Italy; Ajou University School of Medicine, KOREA, REPUBLIC OF

## Abstract

In Italy, Invasive Meningococcal Disease (IMD) due to *Neisseria meningitidis* serogroup B (MenB) showed the highest incidence rates in infants under one year of age. This study describes the main characteristics of MenB responsible for invasive diseases circulating in Italy from 2010 to 2021. Data collected within the framework of National Surveillance System (NSS) for IMD were analysed. Serogroup confirmed IMD cases were included in the study. For unspecified meningococcal serogroup, a multinomial model was used to impute the serogroup. Antimicrobial susceptibility and genotyping by Sanger or whole genome sequencing were performed on viable meningococci. Core genome MLST (cgMLST) was evaluated using a gene-by-gene approach, and MenDeVAR analyses were used to assess potential coverage by MenB vaccines. A total of 1845 IMD cases were reported to the NSS, of which 704 were laboratory confirmed as MenB and another 232 were statistically attributed to this serogroup. The highest incidence rate for IMD due to MenB was observed in infants <1 year, followed by children aged 1–4 years. MenB isolates were susceptible to antimicrobials except for 4 isolates, of which 2 resistant to penicillin G, 1 to rifampicin and 1 to ciprofloxacin. High genetic variability was observed, with clonal complex (cc) cc41/44 being replaced by the cc162 since 2015. MenDeVAR analyses highlighted a high proportion of genomes classified as “insufficient data,” especially among recent isolates. Despite the low incidence of IMD in Italy, MenB increased in specific age groups during the study period. Continuous genomic surveillance, including MenDeVAR monitoring, remains essential to detect changes in circulating MenB and predict vaccine coverage.

## Introduction

Invasive meningococcal disease (IMD) remains a global public health problem, mainly caused by six (A, B, C, W, X and Y) of the twelve recognised *Neisseria meningitidis* serogroups [1]. Traditionally, *N. meningitidis* is associated with severe clinical manifestations such as meningitis, sepsis, or both. However, its clinical spectrum can be broader, including atypical or milder presentations ─ such as pneumonia, pericarditis, epiglottitis, urethritis, gastrointestinal symptoms, pharyngitis, and conjunctivitis─ which can be relatively rare and often unrecognized or underestimated in clinical practice [2,3].

IMD can affects individuals of all age groups and health conditions, although children younger than 4 years of age and patients with specific comorbidities and immunocompromising condition are at highest risk [1–4]. The case fatality rate (CFR) remains quite high worldwide (5–15%), and 20% of survivors may suffer from long-term disability [1–4]. Vaccination and antibiotic treatment (including chemoprophylaxis of close contacts of cases) remain the best strategies for the prevention and control of meningococcal disease [1–4].

Since the introduction of meningococcal vaccines against serogroups A, C, W, and Y, the epidemiology of IMD has changed geographically and temporally, resulting in a shift in the serogroups distribution [1–5]. As a consequence, *N. meningitidis* serogroup B (MenB) has become the main cause of IMD in several European countries, predominantly affecting infants, adolescents and young adults [1–5].

In Italy, meningococcal vaccination policies are defined within the National Immunization Plan (Ministero della Salute, Piano Nazionale Prevenzione Vaccinale. https://www.salute.gov.it/new/it/tema/vaccinazioni/piano-nazionale-prevenzione-vaccinale/ Last visit, 9 July 2025). Possibly, regional differences in vaccine introduction timing, target age groups, and implementation status (routine vs. recommended) need to be considered.

In 2005, MenC vaccination was introduced as recommended at national level, targeting infants and adolescents [6]. Overtime, the MenACYW conjugate vaccine has gradually replaced MenC vaccine and is following the recommendation currently in place for individuals from 12 months of age and for adolescents aged 12–18 years (https://www.salute.gov.it/new/it/tema/vaccinazioni/calendario-vaccinale/).

Regarding to serogroup B, two vaccines ─ the four component meningococcal serogroup B vaccine (4CMenB) and the bivalent factor H-binding protein vaccine (MenB-fHbp) ─ are available in Italy [7]. The 4CMenB vaccine was licensed in 2013 by both the European Medicines Agency (EMA) and the Italian Medicines Agency (AIFA) and it was progressively introduced in the regional immunisation plan [7]. According to official coverage data provided by the Italian Ministry of Health for the period corresponding to this study, vaccination coverage for MenB at 24 months of age was 66.3% in 2020 and 79.7% in 2021, respectively (https://www.salute.gov.it/new/sites/default/files/imported/C_17_bancheDati_38_1_7_file.pdf).

Currently, the 4CMenB is recommended and actively offered free of charge to all infants under one year of age, following a four dose schedule between the 3^rd^ and 15^th^ month of life (https://www.salute.gov.it/new/it/tema/vaccinazioni/calendario-vaccinale/). The bivalent MenB-fHbp vaccine was approved by the AIFA in 2017 and is recommended for individuals aged over 10 years (https://www.aifa.gov.it/sites/default/files/DETERMINA_NP_TRUMENBA_1366.pdf).

The public health measures implemented during COVID-19 pandemic had led to a decline in the circulation of *N. meningitidis*, resulting in a reduction of MenB IMD incidence across the EU/EAA (from 0.3 cases per 100 000 population in 2017 to 0.08 cases per 100 000 population in 2021) [5]. However, MenB remained the predominant serogroup and accounted for 74% of IMD cases in infants aged <1 year in Europe in 2021 [8].

The study aims to describe the epidemiological trend of MenB IMD in Italy from 2010 to 2021 and to characterize the phenotypic and genotypic profiles of invasive strains.

## Materials and methods

### The Italian national surveillance system for invasive meningococcal disease

In Italy, IMD surveillance is part of the National Surveillance System (NSS) for Invasive Bacterial Diseases (IBD) and is coordinated by Istituto Superiore di Sanità (ISS) with the support of the Italian Ministry of Health.

The National Reference Laboratory (NRL) of the ISS receives bacterial isolates and/or biological samples (blood and cerebrospinal fluid) from laboratory-confirmed IMD cases by local laboratories, to perform serogroup identification/confirmation, antimicrobial susceptibility testing and molecular investigations.

For each IMD case, epidemiological information, including patient’s information (age, clinical picture, outcome of disease, and nationality) are routinely collected. Here, data are reported and analysed in a pseudo anonymous and aggregated form. A national report is published annually and made available at https://www.iss.it/sn-mbi-rapporti-iss.

MenB cases refer to both culture and PCR- confirmed cases of invasive meningococcal diseases, collected by NRL of ISS from 2010 to 2021.

The study comprises exclusively invasive meningococcal diseases of serogroup B

### Statistical analysis

Epidemiological data of IMD cases were extracted from the MaBI platform (https://mabi.iss.it/) dedicated to the National Surveillance System of IBD. Variables included in the analysis were: age groups (<1, 1–4, 5–14, 15–24, 25–49, 50–64, ≥ 65 years), seasonality (winter, summer, spring and autumn), and geographical area of residence (North, Centre and South of Italy). Year was included in the models as a categorical variable.

Missing values for serogroup were considered Missing At Random (MAR). As previously described [9], to reclassify missing serogroup values, a multinomial model were used to estimate adjusted rate ratios considering outcome, age group, geographical area, year, and seasonality as predictors.

To assess the predictive power of the multinomial model, a multiclass Area Under the Curve (AUC) was calculated (AUC = 0.79; 95% CI: 0.77, 0.81).

The number of reported cases per 100 000 inhabitants (incidence rate on a yearly basis) will be referred in the text as “incidence”. It should be noted, however, that this value represents the rate of reporting to the system itself, being influenced by the proportion of cases of invasive bacterial disease in which laboratory confirmation and characterization of the etiological agent have been performed.

Crude, age-specific, and age-standardized incidences of MenB were calculated in the dataset with imputed missing data using the Eurostat European population 2010–2021 (Last access: April 8, 2024 https://ec.europa.eu/eurostat/databrowser/product/page/TPS00001).

From 2007 to 2021 it was not mandatory to report the outcome including death of the cases to the surveillance system without obligation to update on the final outcome (i.e., after hospital discharge) as the result of a long-term follow-up and therefore an underestimate of CFR was likely occur Moreover, the serogroup was not defined for all cases.

CFR of IMD by serogroups and binomial exact 95% CI were calculated. CFRs were age-standardized using the Eurostat European population 2010–2021.

IMD cases with missing outcome data (N° = 504) were excluded from this analysis.

Statistical analyses were conducted using R version 4.2.33 (Project for Statistical Computing. Last access: April 8, 2024 https://www.r-project.org/).

### Microbiological analysis

Meningococcal isolates were grown on Thayer Martin agar plate with 2% IsoVitalex (Oxoid, Ltd) incubated in 5% CO_2_ at 37°C. Serogroup identification/confirmation was obtained by slide agglutination with commercial antisera (Remel Europe, Ltd, UK) or by multiplex PCR [10]. Antimicrobial susceptibility to cefotaxime, ceftriaxone, ciprofloxacin, penicillin G and rifampicin was assessed with the Minimum Inhibitory Concentration (MIC) using Etest (Biomerieux, Sweden) and MIC test strip (Liofilchem, Diagnostici, Italy) on Mueller Hinton agar plates (Oxoid, Ltd) supplemented with 5% sheep blood. Clinical breakpoints were those recommended by the European Committee Antimicrobial Susceptibility Testing (EUCAST v. 14.0) [11].

### Molecular analysis

Meningococcal DNA was extracted using QIAamp mini kit (Qiagen, Hilden, Germany), following the manufacturer’s procedure. Species and genogroup identification of biological samples (blood and cerebrospinal fluid) was performed by RT-PCR using MenSerogroup kit (Diagenode, Belgium).

WGS was performed on NextSeq500 or MiSeq sequencers (Illumina, CA, USA). For each isolate 1.5 ng of DNA was used to prepare libraries using the Nextera XT DNA protocol according to the manufacturer’s instructions. The High Output Kit v2 (300 cycles) and the v3 Reagent kit (600 cycles) were used for NextSeq 500 and MiSeq, respectively (Illumina, CA, USA). Quality check of the raw sequence data was performed using FastQC software [12]. High-quality bases (Q score >25) were retained and reads were trimmed using the software Sickle [13]. De novo assembly was performed with ABySS software version 1.5.2 (K parameter = 63) [14].

The *de novo* assembled genomes were uploaded to the BIGSdb platform (https://pubmlst.org/bigsdb), and analysed via a hierarchical gene-by-gene annotation approach. According to the designation tools included in the *Neisseria* PubMLST website (http://pubmlst.org/neisseria/), isolates were characterized by the finetype of two outer membrane proteins – the Variable Region (VRs) VR1 and VR2 of the porin A (PorA) and the VR of the Ferric enterobactin transport (FetA) – and by the Multilocus Sequence Type (MLST). The combination of capsular group, finetype and MLST defines the genotypic profile, as follows: capsular group: PorA (P1). VR1, VR2: FetA (F)VR: sequence type (ST) (clonal complex). Alleles of the main antimicrobial resistance target genes were also analysed: *gyrA*, DNA gyrase subunit A, *penA*, penicillin binding protein 2 (PBP2), and *rpoB*, RNA polymerase β chain.

Genome comparisons were performed on the available MenB genomes collected between 2012 and 2021 using the Genome Comparator tool in the PubMLST database. The resulting distance matrix was visualized as a Neighbor-Net network in Split Tree4 (version 4.13.1). Incomplete loci were automatically removed from the distance matrix calculation for the neighbour-net graphs.

For the culture-negative meningococci, PCR and Sanger sequencing were performed to determine the genotypes, as previously described [15].

The MenDeVAR index [16], a genomic-based tool (available at pubmlst.org/bigsdb?db = pubmlst_neisseria_isolates), integrates genomic information on vaccine antigens with evidence from published serological studies (MATS, MESURE, hSBA) and was used to analyze a subsample of MenB available genomes. As defined, the “exact match” category includes isolates carrying at least one antigen identical to a vaccine variant; “cross-reactive” refers to isolates containing at least one antigenic variant shown in experimental studies to cross-react with vaccine variants, and the “none” category includes variants lacking reactivity in experimental data, while “insufficient data” indicates variants for which experimental evidence is not available.

This study was conducted using data from the National Surveillance System of invasive meningococcal diseases established by law. The samples were collected for the hospitalization routine and no ethical approval was required

## Results

### Invasive meningococcal disease in Italy, 2010–2021

A total of 1845 IMD cases from the original dataset were reported to the NSS from 2010 to 2021 (Last access: May 19 2022. https://mabi.iss.it/). Of all reported cases, 748 (40.5%) were culture-positive, while 1097 (59.5%) were culture- negative and confirmed by molecular methods (PCR and/or Sanger sequencing). Serogroups were identified as follows: 704 cases of MenB, 446 MenC, 79 MenW, 223 MenY, 21 Non Groupable (NG) meningococci, 7 MenX, and 5 MenA. For 360 IMD cases, serogroup information was missing. By multinomial model, they were reclassified as follows: 232 MenB, 116 MenC, 10 NG, and 2 MenY. As shown in Table 1, the frequency distribution by age group, geographical area, year, and seasonality was similar in the original dataset compared to those with imputed missing.

**Table 1:** Comparison between data reported by the National Surveillance System and those obtained by the multinomial model used in this study. Invasive meningococcal diseases (IMD) cases due to serogroup B, by age groups, geographical areas, seasons, and years in Italy, 2010-2021.

	Original dataset ^1^	Adjusted dataset^2^
	MenB IMD cases from National Surveillance System (N° = 704)	MenB IMD cases adjusted by multinomial model (N° = 936)
Age groups (years)	N° of cases (%)	N° of cases (%)
<1	162 (23.0%)	196 (20.9%)
1-4	84 (11.9%)	130 (13.9%)
5-14	89 (12.6%)	127 (13.6%)
15-24	121 (17.2%)	161 (17.2%)
25-49	119 (16.9%)	155 (16.6%)
50-64	73 (10.4%)	95 (10.1%)
≥65	56 (8.0%)	72 (7.7%)
**Geographical area**	**N° of cases (%)**	**N° of cases (%)**
North	406 (57.7%)	509 (54.4%)
Center	181 (25.7%)	204 (21.8%)
South	117 (16.6%)	223 (23.8%)
**Season**	**N° of cases (%)**	**N° of cases (%)**
Winter	271 (38.5%)	364 (38.9%)
Spring	190 (27.0%)	256 (27.4%)
Summer	125 (17.8%)	160 (17.1%)
Autumn	118 (16.8%)	156 (16.7%)
**Year**	**N° of cases (%)**	**N° of cases (%)**
2010	75 (10.7%)	112 (12.0%)
2011	76 (10.8%)	108 (11.5%)
2012	55 (7.8%)	81 (8.7%)
2013	56 (8.0%)	77 (8.2%)
2014	55 (7.8%)	85 (9.1%)
2015	49 (7.0%)	55 (5.9%)
2016	69 (9.8%)	83 (8.9%)
2017	74 (10.5%)	81 (8.7%)
2018	71 (10.1%)	78 (8.3%)
2019	83 (11.8%)	106 (11.3%)
2020	34 (4.8%)	57 (6.1%)
2021	7 (1.0%)	13 (1.4%)

^1^Number of MenB IMD cases obtained from the National Surveillance System (N° = 704)

^2^Number of MenB IMD cases adjusted according to the multinomial model, which imputed a total of 232 out of 360 IMD cases with an unspecified meningococcal serogroup.

**Fig 1:**
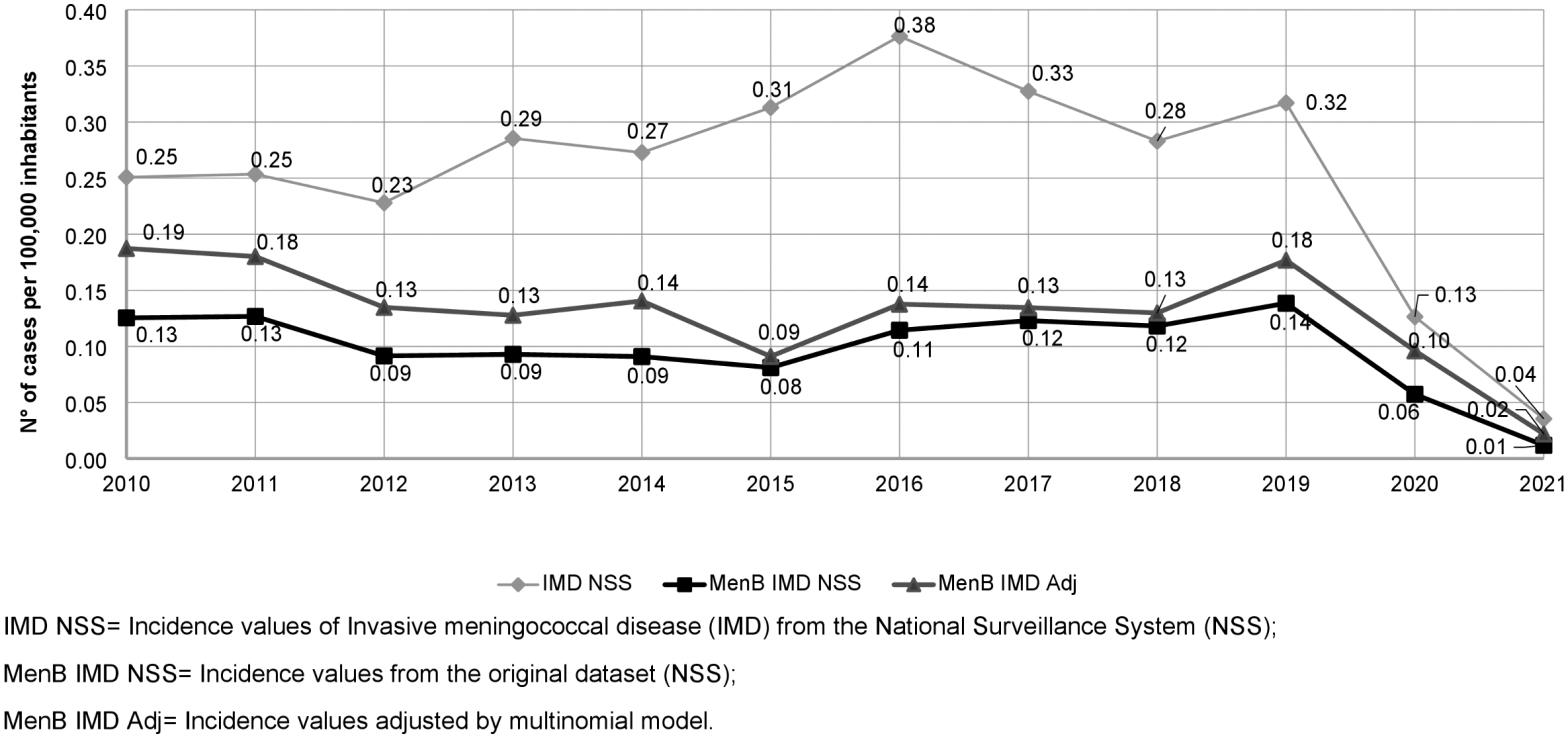
Incidence of invasive meningococcal disease (IMD) caused by serogroup B meningococci (MenB) in Italy from 2010 to 2021. Comparison between incidence values from the original NSS dataset (MenB IMD NSS) and values adjusted by multinomial model (MenB IMD Adj).

Fig 1 shows the difference between MenB incidence calculated using data from the original NSS dataset (here indicated as MenB IMD NSS) and the adjusted values based on the multinomial model (here indicated as MenB IMD Adj). In comparison, MenB IMD Adj values were higher than MenB IMD NSS in the first half of the study period. From 2015, the values were more similar.

During the study period, the highest MenB IMD incidence was recorded in 2019 (0.13 in 2019 per 100 000 inhabitants). In 2020, due to COVID-19 pandemic, the incidence of IMD dropped dramatically, bringing the MenB IMD incidence to 0.06 per 100 000 inhabitants (Fig 1).

**Fig 2:**
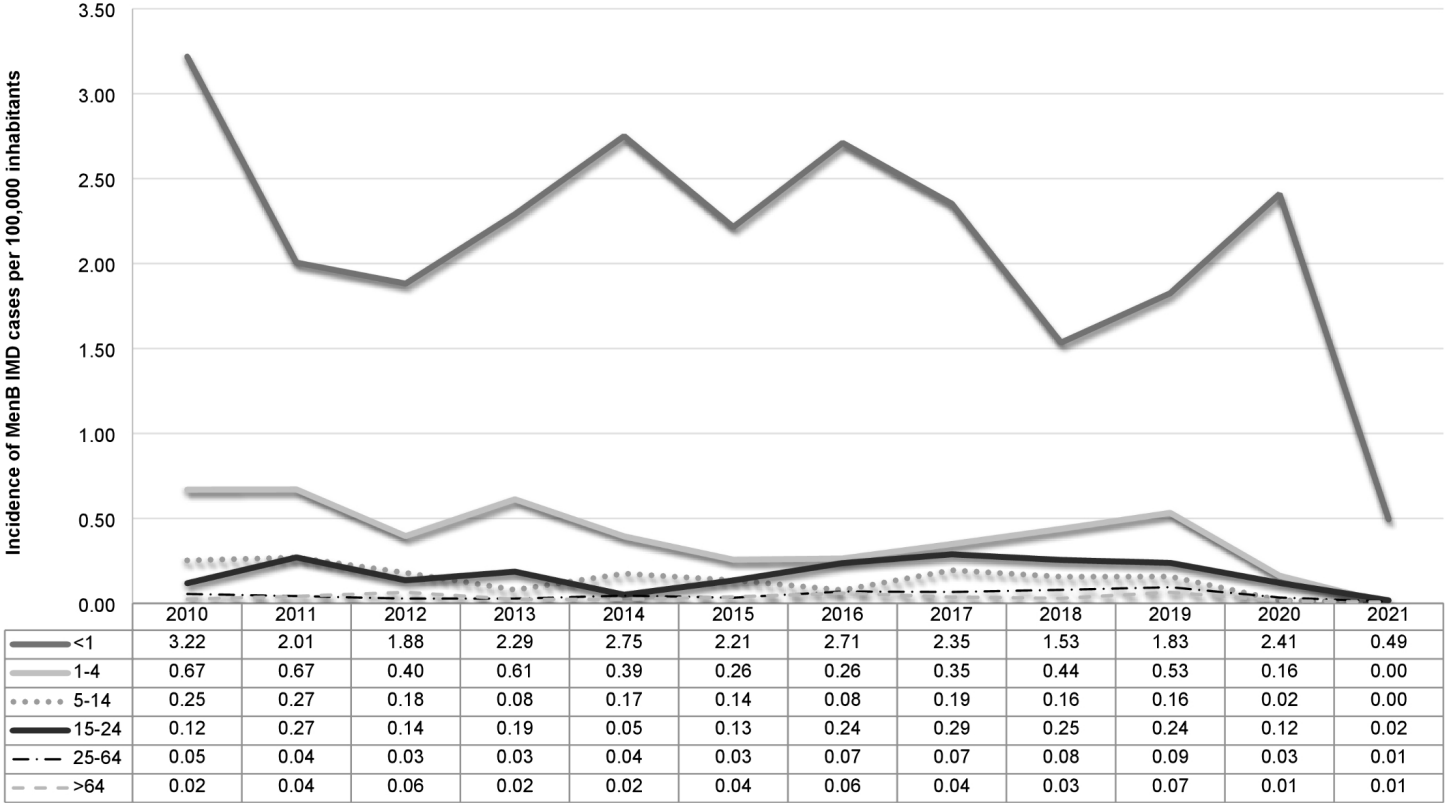
Incidence of invasive meningococcal disease (IMD) caused by serogroup B meningococci (MenB) by age groups in Italy from 2010 to 2021.

Infants aged <1 year followed by children aged 1–4 years were the most affected, showing the highest incidence values during the study period (Fig 2). The lowest incidence was observed in patients aged 25 to ≥ 65 years (Fig 2).

Meningitis was the main clinical presentation (53%; 373/704). Sepsis and sepsis plus meningitis were diagnosed in 46% (321/704) and in 18% (127/704) of patients, respectively.

Other clinical presentations (e.g., pneumonia, arthritis, septic arthritis, peritonitis) occurred in 11 patients.

A total of 504 IMD cases with unknown outcome were excluded from the analysis.

Overall, 203 patients affected by IMD and with a confirmed serogroup died during the study period, of which 64 were due to MenB. The observed CFR for all IMD (obs. CFR) was 15.14% (95% CI: 13.55%− 16.84) while for MenB IMD was 9.29% (95% CI: 7.53%− 11.31) (Table 2). As reported in Table 2, the obs. CFR varied among serogroups; the highest value was reported in IMD due to MenC (26.23%; 95% CI: 7.53%− 11.31) followed by MenW (21.67%; 95% CI: 13.32%− 32.22), NG (11.11%; 95% CI: 2.01%− 31.03), MenY (10.13%; 95% CI: 6.46%− 14.97). For comparison, hereby, the CFR was age-standardized (std. CFR) using the European population. The std. CFR for all IMD was 14.95% (95% CI, 12.96%−17.15) (Table 2), slightly lower than the obs. CFR (15.14%, 95% CI: 13.55%− 16.84).

**Table 2:** Case-fatality rate (CFR) for meningococcal disease by serogroup in Italy, 2010-2021.

Meningococcal serogroup (Men)	Obs. cases	N° of deaths	obs. CFR ^3^	obs.CFR. 95% CI	exp. deaths ^4^	std. CFR ^5^	std.CFR 95% CI
MenA	5	1	20	(1.02 - 65.74)	−	−	−
MenB	689	64	9.29	(7.53 - 11.31)	49.93	10.77	(8.3 - 13.77)
MenC	408	107	26.23	(22.66 - 30.05)	35.97	25.01	(20.5 - 30.23)
NG	18	2	11.11	(2.01 - 31.03)	−	−	−
MenW	60	13	21.67	(13.32 - 32.22)	5.81	18.81	(9.98 - 32.27)
MenX	3	0	0	(0 - 63.16)	−	−	−
MenY	158	16	10.13	(6.46 - 14.97)	12.74	10.56	(6.02 - 17.19)
**Total**	**1341** ^**a**^	**203**	**15.14**	**(13.55 - 16.84)**	**114.18**	**14.95**	**(12.96 - 17.15)**

CFR, case-fatality rate %;

^a^A total of 504 IMD cases with unknown outcome data was excluded from the analysis;

^3^obs. CFR, observed case-fatality rate %;

^4^exp. deaths, number of expected deaths;

^5^std. CFR, standard case-fatality rate calculate using European population (https://ec.europa.eu/eurostat/databrowser/product/page/TPS00001).

For IMD caused by MenB, the std CFR was slightly higher than the obs. CFR (10.77%, 95% CI, 8.3%− 13.77) (Table 2) but lower than other serogroups.

Std CFR was not calculated for Men A, MenX and NG due to the low number of cases (Table 2).

### Antimicrobial susceptibility test and characterization of target genes

Antimicrobial susceptibility was assessed on 322 culture-positive MenB isolates. All isolates were susceptible to cefixime (MIC ≤ 0.125 mg/L) and to ceftriaxone (MIC ≤ 0.125 mg/L). They were also susceptible to penicillin G (MIC < 0.25 mg/L), although 172 MenB isolates showed a MIC values close to the resistance breakpoint (MIC values ranging between ≥0.094 and ≤0.25 mg/L). Two isolates were resistant, with MIC values of 0.38 mg/L for both.

Thirty-seven *penA* alleles were identified, of which *penA14* (N = 31), *penA1* (N = 23) and *penA9* (N = 21), were the most frequent. The 2 penicillin- resistant MenB isolates harboured *penA9* and *penA12,* respectively, and were both characterized by polymorphisms in the C-terminal region of penicillin binding protein 2 (F504L, A510V, I515V, H541N, and I566V).

All isolates were susceptible to ciprofloxacin (MIC ≤ 0.03 mg/L), except for one resistant isolate from 2018 (MIC value of 0.25 mg/L).

Molecular analysis of the target gene *gyrA* identified the following alleles as prevalent: *gyrA*4 (N = 90), *gyrA2* (N = 41) and *gyrA3* (N = 17). For the ciprofloxacin resistant isolate, the presence of the T91I amino acid substitution in the protein sequence, encoded by the *gyrA* gene (allele *gyrA212*) confirmed the antimicrobial resistant phenotype.

MenB isolates were susceptible to rifampicin (MIC < 0.25 mg/L), with the exception of one isolate from 2012, which was resistant to rifampicin (MIC value of 2 mg/L).

Of 15 *rpoB* alleles, *rpoB28* (N = 50), *rpoB18* (N = 30), and *rpoB4* (N = 20) were the most frequent. The remaining alleles (*rpoB1, rpoB2, rpoB7, rpoB9, rpoB31, rpoB34, rpoB38, rpoB40, rpoB72, rpoB73, rpoB85*) were poorly represented.

### Molecular profiles of MenB

A total of 477 MenB were studied at molecular level. Meningococci were grouped into 20 ccs, of which the most common were: cc41/44 (N = 101), cc162 (N = 86), cc32 (N = 49), cc213 (N = 43), cc269 (N = 28), cc461 (N = 23), cc865 (N = 18), cc11 (N = 12), cc1572 (N = 7), (Table 3).

**Table 3:** Genotypic data of the most frequent clonal complexes (cc) of invasive serogroup B meningococci in Italy, 2010-2021.

Clonal complex (cc)	N° of isolates	Sequence Type (ST) (N° of isolates)
cc41/44	101	ST-414 (N° = 12), ST-1403 (N° = 10), ST-1194 (N° = 5), ST-41 (N° = 5), ST-40 (N° = 3), ST-485 (N° = 3), ST-280 (N° = 2), ST-3346 (N° = 2),ST-4742 (N° = 2), ST-6349 (N° = 2), ST-6058 (N° = 2), ST-11851 (N° = 2), ST-43 (N° = 1), ST-154 (N° = 1), ST-1097 (N° = 1), ST-1103 (N° = 1), ST-2719 (N° = 1), ST-3615 (N° = 1), ST-4515 (N° = 1), ST-4759 (N° = 1), ST-5087(N° = 1), ST-5906 (N° = 1), ST-6359 (N° = 1), ST-7151 (N° = 1), ST-8165 (N° = 1), ST-8511 (N° = 1), ST-9504 (N° = 1), ST-9529 (N° = 1), ST-11112 (N° = 1), ST-11119 (N° = 1), ST-11336 (N° = 1),NA (N° = 32).
cc162	86	ST-162 (N° = 63), ST-9293 (N° = 3), ST-8087 (N° = 2), ST-5573 (N° = 1), ST-10175 (N° = 1), ST-10812 (N° = 1), ST-12193 (N° = 1), ST-13870 (N° = 1), ST-9465 (N° = 1), NA (N° = 12)
cc32	49	ST-32 (N° = 4), ST-34 (N° = 4), ST-484 (N° = 1), ST-749 (N° = 1), ST-12031 (N° = 1), ST-2503 (N° = 1), ST-6115 (N° = 1), ST-7460 (N° = 8), ST-8758 (N° = 1), ST-2334 (N° = 1), ST-12392 (N° = 1), ST-33 (N° = 12), NA (N° = 12)
cc213	43	ST-213 (N° = 26), ST-575 (N° = 1), ST-1721 (N° = 1), ST-3496 (N° = 13), ST-7231 (N° = 1), ST-7309 (N° = 2), ST-8955 (N° = 1), ST-9197 (N° = 1), Missing (N° = 7)
cc269	28	ST-269 (N° = 6), ST-467 (N° = 1), ST-479 (N° = 3), ST-1157 (N° = 1), ST-1161 (N° = 1), ST-1163 (N° = 1), ST-1195 (N° = 3), ST-2693 (N° = 1), ST-7610 (N° = 1), ST-8554 (N° = 1), NA (N° = 6)
cc461	23	ST-461 (N° = 6), ST-1946 (N° = 12), ST-3494 (N° = 1), ST-7243 (N° = 1), NA (N° = 3)
cc865	18	ST-3327 (N° = 11), NA (N° = 7)
cc11	12	ST-11 (N° = 10), ST-3537 (N° = 1), ST-8857 (N° = 1)
cc1572	7	ST-1572 (N° = 6), ST-11515 (N° = 1).

NA; sequence data not available.

The remaining isolates belonged to 11 different ccs (cc18, cc23, cc35, cc60, cc167, cc174, cc198, cc334, cc1136, cc1157, cc4821) that were represented by one or two isolates.

For 39 MenB, ccs were not yet assigned according to the current MLST typing scheme (unknown, UNK), while for 47 MenB no genetic information could be obtained.

MenB:cc41/44 represented 31 different STs, where ST-414 (N = 12) and ST-1403 (N = 10) were the most common, followed by ST-1194 and ST-41 (N = 5 isolates each). Other STs were represented by one or two isolates. In contrast, in MenB:cc162, 9 STs were identified. ST-162 was prevalent (N = 63), the remaining STs (ST-5573, ST-8087, ST-9293, ST-9465, ST-10175, ST-10812, ST-12193, ST-13870) were represented by few isolates (Table 3).

Meningococci cc32, cc213, cc269, cc461, and cc865, mainly belonged to ST-32, ST-213, ST-269, ST-1946, and ST-3327 respectively.

Among the main hypervirulent ccs identified, cc11 was found in 12 MenB. In particular, 7 were sporadic cases that occurred between 2011 and 2019; 5 MenB:cc11 constituted an outbreak that arose in Sardinia in 2018 [18].

**Fig 3:**
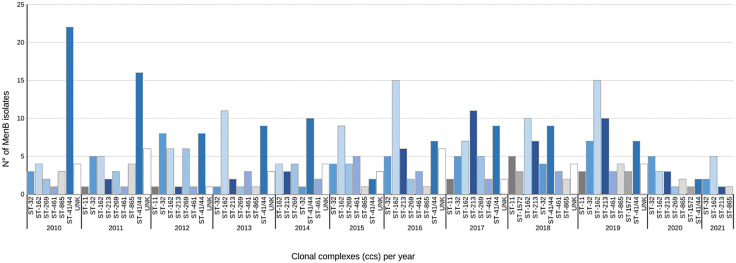
Distribution of the major clonal complexes (ccs) among *Neisseria meningitidis* serogroup B collected in Italy from 2010 to 2021.

Although cc41/44 was the most frequent cc, its prevalence has decreased over time (Fig 3). In contrast, cc162, which accounted for 9% of MenB cases in 2010, increased to 25% in 2019, becoming the predominant cc ─ except in 2017 and 2020, when cc213 and cc32, respectively, were the most prevalent (Fig 3).

A total of 134 finetypes (the combination of PorA VR1, VR2 and FetA VR) were identified, with P1.22,14:F3-6 (N° = 49) in cc162 being the most frequent. Overall, considering the high genetic variability of MenB, the most common genotype was B:P1.22,14:F3-6:ST-162(cc162), representing for 10% (50/477) of the identified genotypes.

**Fig 4:**
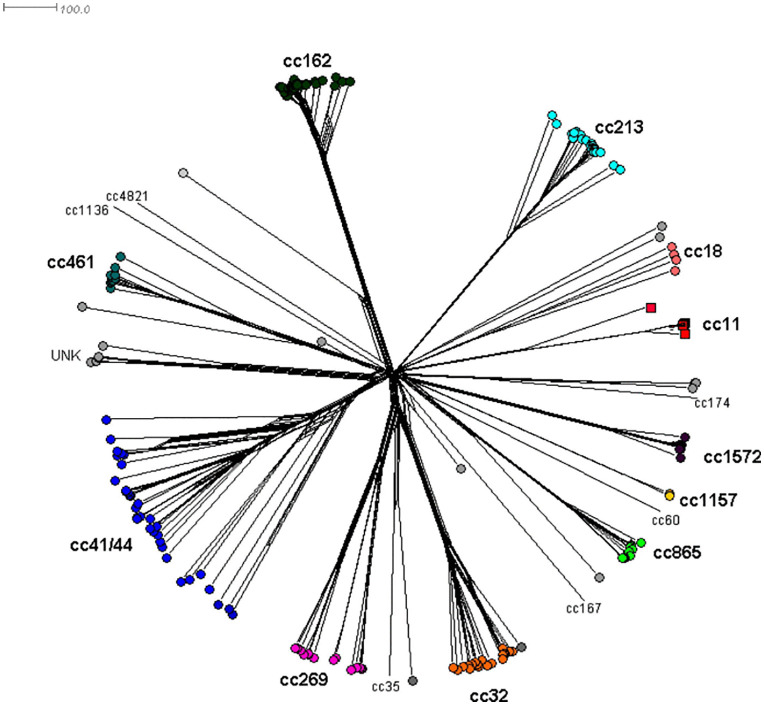
Neighbour-net phylogenetic tree based on core genome MLST (cgMLST) of 201 MenB isolates collected in Italy from 2012 to 2021. Incomplete loci were automatically removed from the distant matrix calculation for neighbour-net graphs. Isolates belonging to the same clonal complex (cc) are highlighted by the same colour.

A comparative genomic analysis was performed on 201 MenB genomes collected between 2012 and 2021 (Fig 4). The phylogenetic tree obtained by cgMLST analysis was reconstructed on the estimated allelic distances and revealed a star-like topology where strains belonging to the same ccs clustered together (Fig 4). Of note, MenB:cc11 isolates (N° = 5) associated with a putative capsule switched strain, from serogroup C to B, and responsible for an outbreak in 2018 [17], formed a single phylogenetic network of the main lineage (Fig 4).

**Fig 5:**
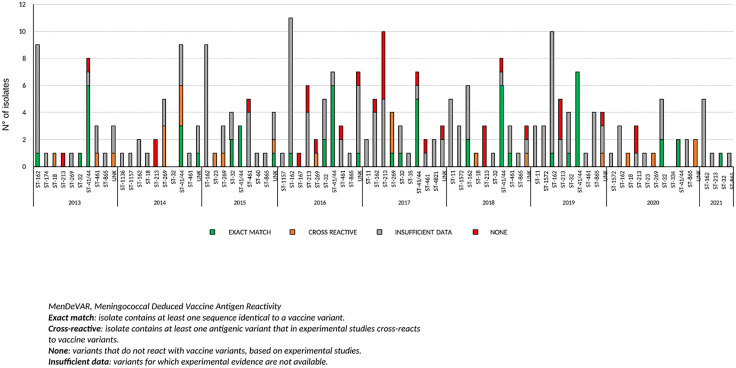
MenDeVAR Index analysis for 4CMenB on 201 meningococcal genomes per year and clonal complexes, 2013-2021.

**Fig 6:**
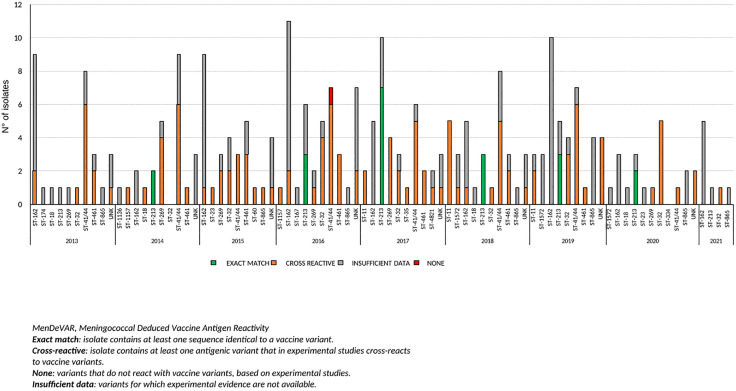
MenDeVAR Index analysis for MenB-fHbp on 201 meningococcal genomes per year and clonal complexes, 2013-2021.

Figs 5 and 6 show the MenDeVAR analyses for 4CMenB and MenB-fHbp, respectively, performed on 201 meningococcal genomes per year and clonal complex from 2013 to 2021.

Among the different clonal complexes, cc41/44 and cc32 showed the highest proportion of genomes with an “exact match” to the vaccine variants in the MenDeVAR analysis for 4CMenB across all years. For most genomes belonging to other clonal complexes, including cc162, the MenDeVAR analysis for 4CMenB indicated “insufficient data”. As previously described, cc213 was the clonal complex with no compatibility (“none” in the MenDeVAR analysis for 4CMenB) due to different antigenic variants (Fig 5).

The MenDeVAR analysis for MenB-fHbp (Fig 6) suggested that cc213 was the only clonal complex with an “exact match” to the vaccine variants. Most genomes carried cross-reactive antigenic variants, with the exception of a single fHbp-negative genome.

For both analyses, “insufficient data” results were obtained for the majority of the genomes.

## Discussion

Hereby, the MenB IMD in Italy from 2010 to 2021 and the phenotypic and genotypic profiles of invasive strains are described. Italy is considered a country with a low incidence of IMD in the overall population [4,5].

MenB has been the predominant cause of IMD in Italy over the last decade, as in many other countries [5]. Exceptionally, during 2015–2016, the incidence of MenC exceeded that of MenB due to an outbreak occurred in Tuscany [18].

Since 2020, preventive measures implemented to reduce the impact of the SARS-CoV-2 pandemic have significantly contributed to the reduction in the incidence of meningococcal disease and MenB cases (0.14 per 100 000 inhabitants in 2019, 0.06 per 100 000 inhabitants in 2020, 0.02 per 100 000 inhabitants in 2021; data available on https://atlas.ecdc.europa.eu/public/index.aspx).

More recently, in 2022, the notification rate for MenB in Italy was 0.06 per 100 000 inhabitants; similar rates were observed in Finland (0.05), Germany (0.09), Greece (0.04), Hungary (0.08), Norway (0.09), Portugal (0.04), and Sweden (0.05) (https://atlas.ecdc.europa.eu/public/index.aspx).

As also reported by other countries [1–4], the incidence of MenB IMD in Italy varied across the age groups.

However, data from the national surveillance system for IMD (2020–2022) [19] described an increase in the percentage of IMD cases caused by MenB in young adults, from 75% in 2021 to 84.6% in 2022 [19]. In contrast, the age group 1–4 years, which was the second most affected age group until 2020, has shown a progressive decline [19].

Regarding CFR by serogroup, a comparison between the observed and age-standardized CFRs, using the Eurostat European population, revealed that the observed CFR by serogroup was slightly higher than the age-standardized CFRs, except for MenB, which showed a lower std. CFR compared to other serogroups. This result is likely due to the differences in population age structure between Italy (with a high proportion of people over 65 to the detriment of infants <1 years of age and adolescents) and other European countries.

Although most IMD cases in Italy were sporadic, several outbreaks, mostly caused by MenC, occurred in our country over the last 20 years [18,20–22]. However, in 2018, an outbreak caused by a capsular switched MenB:cc11 occurred in Sardinia, for which active immunization against MenB was implemented to control the outbreak [17].

In contrast, several MenB outbreaks have been previously reported in Europe and the USA in closed and organization-based settings [23–26]. In Europe, MenB outbreaks were reported in the Republic of Ireland (2010–2013) [25], in France (2012–2013) [24], and in the United Kingdom (2016–2017) [23]. In the USA, the number of MenB outbreaks increased in the last decade [26]. From 2013 to 2018, ten university-based outbreaks caused by MenB were recorded, mainly involving university student communities and their contacts [26]. In these cases, MenB vaccines were used in response to the outbreaks, including chemoprophylaxis for close contacts of case-patients [26].

Antimicrobial susceptibility testing confirmed that MenB isolates were susceptibility to the antimicrobials used for therapy and chemoprophylaxis.

Half of the MenB isolates tested in this study showed penicillin G MIC values close to the resistance breakpoint (ranging between ≥0.094 and ≤0.25 mg/L) and according to the new EUCAST guideline [11], with the exception of 2 resistant isolates, the remaining MenB isolates are considered susceptible to penicillin G.

Consistent with findings in other countries [4], antimicrobial resistant in meningococci remains low in Italy, and associated with sporadic cases.

Molecular and phylogenetic analyses confirm the high genetic variability that distinguishes MenB from other meningococcal serogroups circulating in Italy. Overall, in this study we observed a high number of STs grouped in 20 ccs, of which 5 hypervirulent ccs (cc41/44, cc162, cc32, cc213, cc269) responsible for 65% of MenB IMD cases between 2010 and 2021. Furthermore, compared to other ccs, cc41/44 showed the highest genetic heterogeneity, with 32 STs and 26 finetypes combinations, as also suggested by the phylogenetic analysis.

Of note, molecular characterization revealed a shift in the distribution of ccs over the study period. Cc41/44 was predominant until 2014, then it decreased in favour to cc162, which has become the prevalent in the country. A slight increase of cc213 and cc865 was also observed in the last four years. This scenario was quite different when compared with other European countries where cc269, cc41/44, cc32, cc213, cc461 the main ccs responsible of endemic MenB IMD cases [27] and cc162 was less represented, accounting for only 2.5% [27]. The exception was the Greece, that similarly to our results, showed the cc162 predominant among MenB causing invasive diseases [28]. This situation appears to be the result of multiple factors that need to be taken into account and also suggest the need for further specific studies focusing on the evaluation of functional immunogenicity for this emerging ST, along with integrated analyses combining spatiotemporal data on strain distribution and vaccine coverage. Moreover, suboptimal or heterogeneous vaccine uptake, transmission dynamics, and population movements, may also have contributed to the spread of cc162.

The MenDeVAR index is used to identify potential matches with available vaccine antigens [16].

For 4CMenB, cc41/44, which was one of the most frequent clonal complexes, and cc32 showed the highest proportion of genomes with an “exact match.”

However, it is important to highlight that the MenDeVAR results need to be interpreted in the context of the temporal fluctuations in the prevalence of clonal complexes observed over the years, particularly for cc41/44 and cc162, wich represented the prevalent clonal complexes observed during the study period.

The MenDeVAR analysis for MenB-fHbp indicated that cc213 showed the best correspondence with the vaccine antigens.

Both MenDeVAR analyses classified the majority of genomes as having “insufficient data.”

Overall, these results do not allow a clear definition of the exact match between genomes and vaccine antigens, particularly for the most recently obtained isolates.

Nevertheless, the possibility of evaluating these data *in silico* may be useful for gathering further scientific evidence, especially for genomes classified as “cross-reactive” or “insufficient data.”

From this perspective, strengthening genomic analyses within surveillance systems is essential.

Some limits of the study should be mentioned. Firstly, the data here presented are based on reports from Regions and Autonomous Provinces (PA) submitted to the MaBI platform for invasive bacterial diseases voluntary. Information on individual vaccination status in the MaBi platform is not often systematically reported. Secondly, the surveillance system only includes laboratory-confirmed IMD cases, and molecular characterization was performed on most, but not all, meningococcal isolates. As a result, genomic information was available only for a subset of IMD cases. Finally, the outcome of each IMD case is mandatory since 2022 and without obligation to update on the final outcome (i.e., after hospital discharge) as the result of a long-term follow-up. This could lead to an underestimation of CFR by serogroup.

In conclusion, MenB remained the predominant cause of IMD in Italy during the study period. Molecular investigations of genotypic profiles highlight the dynamic nature and genetic diversity of this serogroup, which continues to evolve. Molecular monitoring is essential to identify the emergence of new variants with specific virulence characteristics.

## Supporting information

S1 Table Accession numbers of *Neisseria meningitidis* genomes included in this study. (XLSX)
